# The Effect of Music as a Non-Pharmacological Intervention on the Physiological, Psychological, and Social Response of Patients in an Intensive Care Unit

**DOI:** 10.3390/healthcare11121687

**Published:** 2023-06-08

**Authors:** Magdalena Lorek, Dominika Bąk, Katarzyna Kwiecień-Jaguś, Wioletta Mędrzycka-Dąbrowska

**Affiliations:** 1 Student Scientific Club of Anesthesia and Intensive Care, Medical University of Gdansk, 80-211 Gdansk, Poland; magda99@gumed.edu.pl (M.L.); dombak@gumed.edu.pl (D.B.); 2 Department of Anesthesiology Nursing & Intensive Care, Faculty of Health Sciences, Medical University of Gdansk, 80-211 Gdansk, Poland

**Keywords:** intensive care unit, music perception, music therapy, classical music, delirium, pain, therapeutic music listening, nursing

## Abstract

Introduction: Music is an intriguing but relatively under-researched intervention with many potential benefits for mechanically ventilated patients. The review aimed to assess the impact of listening to music as a non-pharmacological intervention on the physiological, psychological, and social responses of patients in an intensive care unit. Methods: The literature review was conducted in the fourth quarter of 2022. The overview included papers found in Science Direct, EBSCO, PubMed, Ovid, Scopus, and original research papers published in English meeting the PICOS criteria. Articles published between 2010 and 2022 meeting the inclusion criteria were included for further analysis. Results: Music significantly affects vital parameters: decreases the heart rate, blood pressure, and breathing; reduces pain intensity. The analyses confirmed that music affects anxiety levels, reduces sleep disturbances and delirium occurrence, and improves cognitive function. The effectiveness of the intervention is influenced by the choice of music. Conclusions: There is evidence of the beneficial effects of music on a patient’s physiological, psychological, and social responses. Music therapy is highly effective in reducing anxiety and pain and stabilizes physiological parameters, i.e., the heart rate and respiratory rate, after music sessions in mechanically ventilated patients. Studies show that music reduces agitation in confused patients, improves mood, and facilitates communication.

## 1. Introduction

Music is an intriguing but relatively under-researched intervention with many potential benefits for mechanically ventilated patients in an intensive care unit (ICU) [1]. During the treatment process, patients experience circadian rhythm changes and sleep disturbances and are at a high risk of delirium, an acute brain injury syndrome [2,3]. An intubated patient experiences pain, anxiety and physiological stress, fear of death, changes in the environment, and restriction of movement due to invasive and non-invasive monitoring [4]. The American Music Therapy Association defines music therapy as the use of personalized listening to music as a therapeutic tool by healthcare professionals, which can be conducted by a nurse or a nursing assistant after brief training [3,5,6,7].

Listening to music can be treated as a cheap, non-invasive, non-pharmacological method of reducing anxiety in patients. Music can have an analgesic effect, among others, by increasing the secretion of endorphins, peptide hormones produced in the central nervous system. Endorphins, in addition to relieving pain, also provide a feeling of bliss and euphoria [8,9]. It is used to reduce stress and anxiety and relieve pain, nausea, delirium, and depression [8]. Music can provide a balance between the mind, body, and soul. According to numerous studies, music experiences are effective and practiced as an integrative non-pharmacological intervention to support medicine by ICU nurses [8,9]. Without a doubt, nurses should be equipped with the clinical competences to meet patients’ needs and families’ expectations [10]. Recently, in nursing, music was identified as one of the contextual factors that can positively influence patients’ clinical outcomes by stimulating placebo effects and avoiding nocebo effects. The ideal example of this is when clinical nurses can improve patients’ symptoms and well-being by creating a comfortable environment [11,12,13]. Music can trigger emotional responses that improve the quality of life, but by the same token, music can also induce stress and aggressiveness. The use of complementary intervention improves concentration and also affects neuropsychological aspects, the effectiveness of which depends on individual preferences [14]. Some researchers recommend using original music to avoid the risk of negative emotions in patients and unpleasant memories after discharge from the hospital. Others, mainly in the perioperative period, advocate that providing music familiar to the patient enhances the positive emotional effects induced by listening [9]. The highest health benefits for intensive care patients are seen in the use of classical music. Some research suggests that classical and meditative music activate the left and right hemispheres of the brain. The simultaneous operation of the left and right hemispheres maximizes learning and remembering information. Heavy metal or techno music are not only ineffective but potentially dangerous and can lead to stress and/or life-threatening arrhythmias, especially in ICU patients [14]. Music tracks should have a slow rhythm, 60–80 beats/min., and be in sync with body rhythms; a calm environment should be provided with restricted light or eye mask usage [9]. The available analyses show that the issues of music therapy and medicine in the area of intensive care are still not fully understood. First, it is important to distinguish music therapy from musical intervention as a method of patients listening to music. Secondly, there is still not enough knowledge about how music improves the well-being of patients. Many of the analyzed studies have shown that this non-pharmacological intervention is very often carried out incorrectly. Thirdly, musical interventions for critically ill patients can promote rapid recovery from disease, but the inappropriate use of music can also increase anxiety and depression.

### Aim

The review aimed to assess the impact of listening to music as a non-pharmacological intervention on the physiological, psychological, and social responses of patients in an intensive care unit.

## 2. Methods

### 2.1. Study Design

The literature review was conducted in the fourth quarter of 2022. This review was prepared on the recommendations of the Prisma 2020 Guidelines for scoping reviews [15,16]. Scoping reviews, a type of knowledge synthesis, follow a systematic approach to map evidence on a topic and identify the main concepts, theories, sources, and knowledge gaps [15].

### 2.2. Search Methods

The following databases were searched: Science Direct, EBSCO, PubMed, Ovid, and Scopus. The keywords used were “music”, “pain”, “intensive care”, “delirium”, or combinations of these using AND or OR operators. A total of 255 articles matching the primary search criteria were found, 160 of which were included in the further analysis, which included the verification of the availability of full-text versions and their compliance with the inclusion criteria. The last search was conducted on 8 January 2023. Finally, 18 articles published from 2010 to 2022 were submitted for our review. The review and qualification of articles for further analysis were possible after meeting the inclusion criteria.

### 2.3. Inclusion and Exclusion Criteria

**Figure 1:**
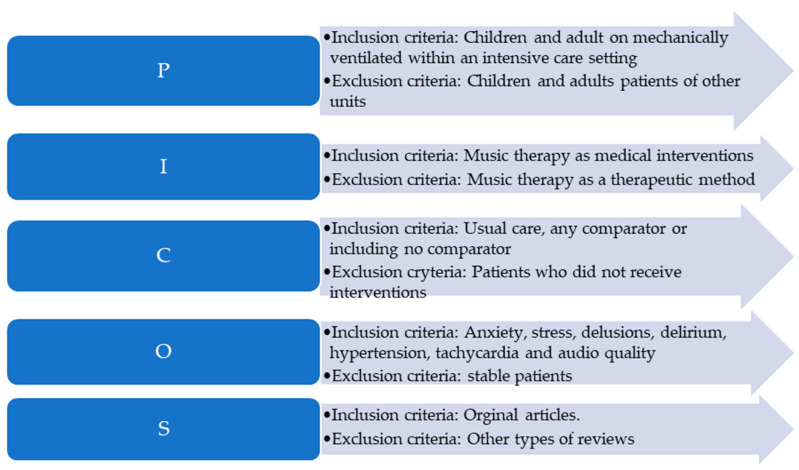
Inclusion and exclusion criteria (PICOS).

Papers published in English were included in the analysis. The inclusion and exclusion criteria were based on the PICOS classification and are further detailed in Figure 1.

### 2.4. Data Extraction

Qualified articles meeting the inclusion criteria were analyzed by an independent reviewer with the following criteria: author, date of publication, the aim of the study, sample (study and control group), materials and methods, results, and implications for nursing practice. Finally, 18 qualitative articles meeting the PICOS criteria from two independent searches were included for further analysis.

### 2.5. Ethical Aspects

The consent of the bioethical commission was not needed to conduct a literature review due to the type of article.

### 2.6. Assessment of the Study Quality of the Included Studies

The screening of titles and abstracts was completed independently, then in duplicate, by two authors (M.L., D.B.). Any studies appearing to meet the inclusion criteria were retrieved as full-text articles. Two reviewers then read the full-text articles in their entirety to assess for eligibility, with decisions on inclusion and exclusion recorded according to the PICOS classification. Any disagreements were discussed with another reviewer.

In the next step, two reviewers (M.L., D.B.) independently performed quality appraisals using the tool developed by the Joanna Briggs Institute (JBI) [17]. The JBI’s approach to evidence-based healthcare is unique. The methods developed by the JBI were designed to provide authors with a comprehensive guide on how to conduct a systematic review and how to evaluate selected articles (JBI for manual synthesis https://jbi-global-wiki.refined.site/space/MANUAL, 15 December 2022). For this purpose, the JBI Critical Appraisal Checklist was used, which provides a checklist with 11 criteria (Q1–Q11). The questions in the checklist focused on the inclusion criteria of selected articles, the sources and resources of selected material, and what kind of methods were used in the study. The answers used are yes, no, unclear, or not applicable. The results of this evaluation are presented in Table 1. The results of the discussion were used as the final results for the quality appraisal of the included studies. 

### 2.7. Data Analysis

The synthesis was carried out and presented graphically and as tables as appropriate. No meta-analysis was carried out.

### 2.8. Selection of the Source of Evidence

The review process was divided into three phases: 1. searching for relevant manuscripts using the search strategy in different databases; 2. including or excluding articles based on their abstracts and inclusion criteria; 3. checking for article relevance in full-text articles and preparing the literature review.

## 3. Results

**Figure 2:**
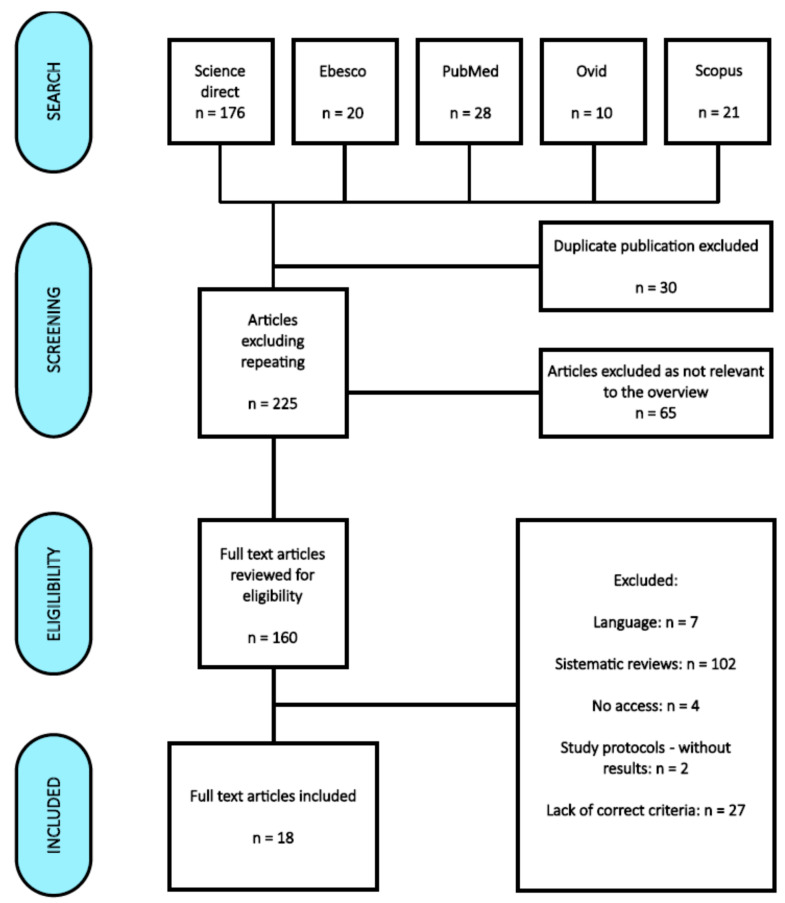
PRISMA flow diagram [8].

A total of 256 publications were initially obtained from the databases: Science Direct—176, EBSCO—20, PubMed—28, Ovid—10, and Scopus—22. After discarding duplicates and selecting titles and abstracts, 128 were excluded. Ultimately, 18 articles were selected for review [3,7,9,18,19,20,21,22,23,24,25,26,27,28,29,30,31,32]. The results are presented in Figure 2.

Of the 18 included studies, 13 (72.22%) of them were reports of randomized controlled trials or pilot-controlled trials. One paper was a pilot study, and one was a cross-sectional intervention. Other studies were based on the pretest and post-test design. The last study included in the systematic review was a retrospective cohort study.

The literature review was aimed at investigating and presenting the current state of knowledge on supporting the treatment of ICU patients with music based on scientific publications. Table 2 presents a summary of the main results of the review. The last literature review presenting an analysis of articles approximating the topic of music therapy in an ICU was published in 2010 [18]; thus, one of the inclusion criteria was the year of publication. Subsequent studies provide an opportunity to better understand the effect of music on the patient, as its impact is still unrecognized [19,20].

A detailed synthesis of the qualitative findings is presented in Table 2.

### 3.1. Demographic Data

Most of the included studies were conducted in the United States of America (*n* = 9). Other countries mentioned in the included articles were: India, Australia, Taiwan, Switzerland, Columbia, Turkey, China, Iran, and France.

### 3.2. Characteristics of the Study Population

A total of 1598 participants were included in 18 studies. One study focused on relatives of ICU patients. The mean age of the patient population included in the study was 60 years old. In seven manuscripts, the authors did not provide detailed information about the patient’s age. Scales that were used to assess patient reactions to music intervention were: the Richmond Agitation and Sedation Scale (*n* = 4), the Likert scale (*n* = 2), and the Glasgow Coma Scale (*n* = 3). Other scales were: the Chinese version of the Spielberger State-Trait Anxiety Scale, the Hospital Anxiety and Depression Scale (HADS), the Facial Anxiety Scale, the Critical Care Pain Observation Tool, the Numeric Rating Scale, and the Visual Analog Scale. Six of the eighteen included articles (33.3%) indicated that nursing staff could and should include patients listening to music in their nursing interventions (Table 2).

### 3.3. Types of Music Therapies

It is important to distinguish between forms of treatment with music because therapeutic music listening is very often confused with music therapy, as these two terms are not the same. “Therapeutic music listening should not be confused with music therapy”. The first form of music used in the form of music therapy can only be conducted by a certified music therapist, while therapeutic music listening is conducted by nurses, among others, through patients listening to music on headphones or volunteers playing live music [21]. Golino et al., 2019, in their publication, present a juxtaposition of the terms music therapy and music listening [3]. Browning et al. and Buzzi et al. point out that therapeutic music listening can be conducted by staff without training since such training in order to play music to patients is not necessary. The authors also found that playing music to patients from a player is far less expensive than using individual headsets for each patient [29,33].

### 3.4. The Impact of Music on the Physiological, Psychological, and Social Responses of Patients in an Intensive Care Unit

Studies by Golino et al., Seyffert et al., Khan et al., Ettenberger et al., and Johnson K. et al. indicate that music has a significant effect on vital signs and decreases the heart rate, blood pressure, and respiration [3,19,21]. On the other hand, Ettenberger et al., Lee et al., and Johnson et al. confirmed that music can reduce pain and anxiety levels [21,23,24], as well as reduce sleep disturbances [21,25] and have a relaxing effect and regulate breathing [22]. Studies by Dallı et al., Browning et al., and Jawaharani et al. emphasize that music therapy reduces the incidence of delirium [25,29,30], and Yekefallah et al. pointed out that it can improve cognitive function [32]; in addition, it can result in reduced costs of drug treatment [26]. Some studies have shown that music therapy has a positive effect on vital signs, except for diastolic pressure [20,23,31], and can accelerate heart rate [20]. Cousin et al. also proved that music helps young patients (children) communicate better, and all subjects showed improvement in coping with stress during hospitalization [31]. The effectiveness of interventions is influenced by the choice of music [31]. The available analysis also shows that there are studies that do not indicate that music therapy in an ICU has any benefit for patients [27,28].

## 4. Discussion

The analysis of selected articles made it possible to present the impact of music on the physiological, psychological, and social spheres of life of patients from intensive care units with various ailments, at different ages, with the use of different forms of treatment with the help of music. In everyday life, medical teams strive to improve patient care. Focusing on the patient is of crucial importance in the quality of care, especially since post-ICU symptoms such as anxiety, depression, or post-traumatic stress disorder (PTSD) may occur up to 90 days after an ICU stay [9]. Music therapy is a new therapeutic tool accepted by most patients [34,35]. However, before music therapy is applied, attention should be paid to the available forms of therapy and the possibilities of their implementation in the hospital ward. The analyzed publications indicate that therapeutic music listening is very often confused with music therapy, and these are not the same [3,21]. Only therapeutic music listening can be implemented by nursing personnel [21].

Types and forms of music therapies

Although therapeutic music listening can be used in everyday clinical practice and has beneficial effects for ICU patients of all ages, it is not always used. Various forms of using music were used in the researched original papers. In studies by Cooke et al., Lee et al., and Johnson et al., patients listened to music on headphones connected to a portable CD player or an MP3 player [23,24,27]. Some authors confirmed that music should have a slow tempo and low volume because the processes taking place in the brain during its playback have a calming effect [23]. In addition, Khan et al. used three different forms of supporting the patients’ treatment and showed that slow-tempo music was the best [20].

The choice of musical instruments is also important. Chiasson et al. conducted a music therapy session using a harp played by a professional harpist [28]. In the study by Cousin et al., a certified music therapist used different instruments, including the allophone, ukulele, and kalimba [31]. Playing live music on musical instruments requires the employment of qualified musicians or music therapists, which is associated with additional costs. As noted by Browning et al. and Buzzi et al., 2022, music played on a device by the ward staff does not require training and is a cheaper solution [29,33]. The use of this form of musical intervention also has a positive effect on improving the patients’ vital parameters: patients listening to music with a slow tempo on headphones had an improvement in heart rate and systolic blood pressure [24]. Patient participation in the musical intervention also affects decreases in self-reported pain and respiratory rate [3].

The impact of music on the physiological, psychological, and social responses of patients in an intensive care unit.

Supporting treatment with the use of music applies to patients in various clinical conditions. A specific musical intervention conducted in the study by Buzzi et al. by musicians and music therapists decreased the heart rate in pediatric patients [33]. Music therapy interventions may also reduce anxiety and pain in critically ill children [31].

The results of the collected studies often do not show a statistically significant effect on the improvement of a patient’s condition. The lack of dependence may be due to the small size of the study group. Despite this, some publications showed a positive impact of music on the physiological sphere of patients’ lives by improving vital parameters such as heart rate, respiratory rate, anxiety levels, blood pressure, and pain [3,20,24]. Han L. et al. showed that a music session stabilized vital signs in invasively ventilated patients [18]. One 20–30 min session of musical intervention can relieve pain in patients [36]. According to Cousin et al., music therapy is a method that can be successfully used in an intensive care unit and is accompanied by a high level of recognition not only by the families of patients staying at the ICU but, above all, by the staff working there [31].

The second issue is that there are still unrecognized ways to measure/test a patient’s mental or social sphere. The effects of music on the mental and social spheres have been assessed based on patients’ reported and observed reactions/parameters: better communication, calmness or agitation, coping with stress, and alertness [30,31].

A study by Chahal et al. found that 85% of patients admitted to an ICU experienced anxiety, and similarly, Castillo et al. showed that 82% of patients experienced anxiety, but the music therapy used was highly effective in reducing anxiety and stabilizing physiological parameters among those in the experimental group at post-test compared to the conventional social care group, which received no intervention beyond routine care [37,38]. Han et al. showed that even a single session of music significantly reduced anxiety in patients compared to those who did not receive music [18]. Mechanically ventilated patients who had music played on headphones by nurses had reduced stress and anxiety [23].

Patient participation in guided music therapy reduces pain, lowers elevated vital signs, and speeds up recovery processes after discharge from intensive care. When it comes to music cognition, the current results are particularly relevant to studies on aesthetic preferences, style or genre preferences, and musical choices [39]. The brain activation data revealed that broad limbic and paralimbic regions related to emotion, as well as reward circuits, are significantly more active for familiar music compared to unfamiliar music. Smaller areas in the cingulate cortex and frontal lobe, including the motor cortex and Broca’s area, were found to be more active in response to liked versus disliked music. Therefore, familiarity appears to be a key factor in listeners’ emotional engagement with music, as shown by the fMRI data [40].

Recent research on empathy and aggression from a music therapy perspective highlights the importance of participants’ subjective worldviews when it comes to experiencing empathy. Similarly, the influence of music therapists’ spiritual or religious beliefs on their perception of empathy further complicates the situation, indicating that divergent meanings of empathy can be found not only among healthcare users but also among healthcare professionals [41].

## 5. Limitations

The study has several limitations. First was the methodological quality of the presented research. All included articles were original studies and met the PICOS inclusion criteria. Unfortunately, after the evaluation process, we can say that there was no standardized tool to measure the effect of music therapy on patients’ well-being. Some researchers used the Glasgow Coma Scale, others the CAM-ICU, CPOT scales, or physiologic parameters. This was the main difficulty in comparing and generalizing the results. Another barrier refers to the intervention used. Some authors used music therapy, while other research projects focused on patient-direct music therapy (PDMT). Music sessions in the articles included in the review process last from 15 to even 60 min, which differs from the American Music Therapy Association recommendations. All patients included in the studies were adults; the study groups of individual components were not differentiated. In some publications, the presence of a control group was indicated. In other observations, the research had a more qualitative nature.

The last limitation was that this review only included studies available in English, and the review could be replicated in other languages for comparison and wider representation.

## 6. Implications for Nursing Practice

Influencing the patient with music is not an entirely new method in medicine, although, in an intensive care unit setting, this form of influencing the patient is a return to the tenets of holistic medicine, that is, one that combines the work of the body with the work of the mind. Analyses of available studies have confirmed the benefits of therapeutic music listening.

The music played, to bring the best therapeutic benefits, should be a slow tempo with a low volume, not exceeding 65 dB. Many authors confirm that therapeutic music listening can and should be incorporated into nursing interventions as part of non-pharmacological treatments for both pain and delirium. Contrary to appearances, the use of this method does not require specialized, expensive equipment or many nursing staff. All that is required is a short training course, access to a radio, and scheduling a 20–30 min music session that can be repeated four times a day. Nursing staff who would like to incorporate a therapeutic music listening program can refer to the American Music Therapy Association’s detailed rules and guidelines for conducting such activities. The association recommends that during ongoing music sessions, when evaluating the effectiveness of ongoing activities, staff should closely observe patients’ behavior and responses, including the presence of tears, physical responses (movement), verbal expression, eye contact, and changes in breathing or muscle tone. Only by carefully observing and noting changes in the patient’s observation chart will there be a real opportunity to assess the effectiveness of the activities carried out. Undoubtedly, it is worthwhile to deepen the topic of the impact and benefits of using music in the intensive care unit setting, especially since the use of therapeutic music listening as one of the few intervention methods is very well-received by the medical community, but also by the families of patients.

## 7. Conclusions

Analyses have given evidence of the beneficial effects of music on the physiological, psychological, and social responses of patients. Music therapy is highly effective in reducing anxiety and pain and stabilizing physiological parameters, i.e., the heart rate and the respiratory rate in mechanically ventilated patients. Studies show that music reduces agitation in confused patients, improves mood, and facilitates communication. Conducting music therapy is very well-received not only by staff working in an intensive care unit but also by family members who visit patients.

## Figures and Tables

**Table 1:** Critical appraisal results for included studies.

Author, Year	Q1	Q2	Q3	Q4	Q5	Q6	Q7	Q8	Q9	Q10	Q11
Golino et al., 2019 [3]	Y	Y	Y	Y	Y	Y	Y	Y	Y	Y	Y
Heiderscheit et al., 2022 [7]	Y	Y	Y	Y	Y	Y	n/a	Y	U	Y	Y
Messika et al., 2026 [9]	Y	Y	n/a	Y	Y	Y	n/a	Y	U	Y	Y
Han et al., 2010 [18]	Y	Y	Y	Y	Y	Y	n/a	Y	U	Y	Y
Seyffert et al., 2022 [19]	Y	Y	Y	Y	Y	Y	n/a	Y	U	Y	Y
Khan et al., 2020 [20]	Y	Y	Y	Y	Y	Y	Y	Y	N	Y	Y
Ettenberger et al., 2021 [21]	Y	Y	Y	Y	Y	Y	n/a	Y	U	Y	Y
Ames et al., 2017 [22]	Y	Y	Y	Y	Y	Y	n/a	Y	U	Y	U
Lee et al., 2016 [23]	Y	Y	Y	Y	Y	Y	Y	Y	Y	Y	Y
Johnson et al., 2018 [24]	Y	Y	Y	Y	Y	Y	Y	Y	N	Y	Y
Dallı et al., 2022 [25]	Y	Y	Y	Y	Y	Y	n/a	Y	U	Y	Y
Chlan et al., 2019 [26]	Y	Y	Y	Y	Y	Y	n/a	Y	U	Y	Y
Cooke et al., 2010 [27]	Y	Y	Y	Y	Y	Y	Y	Y	N	N	Y
Chiasson et al., 2013 [28]	Y	U	Y	Y	Y	Y	Y	Y	Y	Y	Y
Browning et al., 2020 [29]	Y	Y	Y	Y	Y	Y	Y	Y	Y	Y	Y
Jawaharani et al., 2020 [30]	Y	n/a	Y	Y	Y	Y	Y	Y	N	Y	Y
Cousin et al., 2022 [31]	Y	n/a	Y	Y	Y	Y	Y	Y	N	Y	Y
Yekefallah et al., 2021 [32]	Y	Y	Y	Y	Y	Y	n/a	Y	U	Y	Y

Y—Yes, N—No, U—Unclear, n/a—not applicable Q1: Was the review question clearly and explicitly stated? Q2: Were the inclusion criteria appropriate for the review question? Q3: Was the search strategy appropriate? Q4: Were the sources and resources used to search for studies adequate? Q5: Were the criteria for appraising studies appropriate? Q6: Was the critical appraisal independently conducted by two or more reviewers? Q7: Were there methods to minimize errors in data extraction? Q8: Were the methods used to combine studies appropriate? Q9: Was the likelihood of publication bias assessed? Q10: Were recommendations for policy and/or practice supported by the reported data? Q11: Were the specific directives for new research appropriate?

**Table 2:** Synthesis of qualitative findings for a literature review in the field of music and medicine.

Author and Date	Aim	Sample		Materials and Methods	Results	Implication for Nursing Practice
		Study Group	Control Group (CG)			
Golino et al., 2019 [3]	Impact of an active music therapy intervention on intensive care patients	Adult patients		- Pretest–post-test, within-subject, single-group design - 30 min music therapy session (relaxation intervention or a song choice intervention) - Measuring vital signs before and after	After the intervention, differences were found in the following vital functions: respiratory rate, heart rate, pain, and anxiety levels	Music therapy can be a form of non-pharmacological nursing intervention. It responds to the patient’s individual needs without posing risks to the patient
		52	X			
Seyffert et al., 2022 [19]	Decreasing delirium through music listening (DDM) in critically ill, mechanically ventilated older adults in the intensive care unit: a two-arm, parallel-group, randomized clinical trial	160 mechanically ventilated adults		- Randomized controlled trial - 1 h music listening sessions twice daily - 1 h sessions of a silence track twice daily - Patient Health Questionnaire-9 (PHQ-9) - Generalized Anxiety Disorder-7 (GAD-7)	Decreases in heart rate and blood pressure	Music listening has been shown to activate areas of the brain involved with memory, cognitive function, and emotion
		80	80			
Khan et al., 2020 [20]	Decreasing delirium through music	52 patients on mechanical ventilation		- A randomized pilot trial - Group listening to personalized music, slow-tempo music, audiobooks - The intervention lasted one hour, twice a day for about 7 days - Patients wore noise-canceling headphones and used mp3 players - Assessment of delirium twice a day according to a scale (CAM-ICU)	- 80% of patients surveyed rated the music as enjoyable - Patients in the STM group had more median delirium/coma-free days by day 7 - Patients in the STM group had significant increases in heart rate and diastolic blood pressure compared with other groups of patients	The use of music intervention in the intensive care unit is associated with reduced patient anxiety and, thus, less frequent use of sedative drugs. However, it can pose some logistical challenges in a nurse’s work
Ettenberger et al., 2021 [21]	The effect of music therapy on perceived pain, mental health, vital signs, and medication usage of burn patients hospitalized in the intensive care unit	Adult burn patients from an ICU		- Randomized controlled trial (RCT) protocol with two parallel arms - Relaxation through music therapy, music-assisted relaxation (MAR) protocol - Assessment of the pain level on the VAS scale before and after intervention, measurement of vital signs - Estimated hospitalization of >7 days	- Efficacy of action in the occurrence of sleep disorders and the reduction of agitation - MAR could modulate the expression of particular frequency rhythms having an important role in reducing the network activity implicated in pain perception	The mechanisms of live and paired music can affect pain perception in adult burn patients
Ames et al., 2017 [22]	Music listening among postoperative patients in the intensive care unit	41 surgical patients from an ICU		- A randomized controlled trial with mixed-methods analysis - Patients during opioid treatment - Patients were evaluated according to the NRS and VAS scales (before and after intervention) - Music therapy lasted about 50 min, at least 4 times a day - The patients’ stay in the intensive care unit was 48 h	The study found no significant effect on opioid use. The NRS score was found to be lower in the intervention group, and patients’ well-being also improved	Music therapy can be an additional intervention used by nurses for medical treatment, which has no side effects on the patient and can have a positive impact on pain sensations
		20	21			
Lee et al., 2016 [23]	Effects of music intervention on state anxiety and physiological indices in patients undergoing mechanical ventilation in the intensive care unit	85 patients admitted to the ICU		- A randomized controlled trial - Patients individually listened to music from 4:00 to 4:30 p.m. - Control group patients wore headphones but heard no music for the same 30 min - The Chinese version of the State-Trait Anxiety Inventory, the Visual Analog Scale for Anxiety, heart rate, and blood pressure	After adjusting for demographics, an analysis of covariance showed that the music group had significantly better scores for all post-test measures and pre-post differences (except for diastolic blood pressure)	A 30 min intervention can already produce beneficial effects for the patient, and due to its low cost and ease of application, it can be used by nurses to reduce patient anxiety. Before and after the intervention, the nurse should monitor the patient’s condition
		41	44			
Johnson et al., 2018 [24]	Music intervention to prevent delirium among older patients admitted to a trauma intensive care unit and a trauma orthopedic unit	40 patients aged >55 and older		- Randomized controlled trial - Intervention for 60 min, twice a day, at 2 p.m. and 8 p.m. for a period of three days after admission - SBP, DBP, HR, and BP measurement - Pre-recorded self-selected music using an iPod and headsets with a slow tempo	Statistically significant differences in heart rate pre-/post-music listening	No data
		20	20			
Dallı et al., 2022 [25]	The effect of music on delirium, pain, sedation, and anxiety in patients receiving mechanical ventilation in the intensive care unit	36 patients		- A single-blind, randomized, controlled trial - Assessment scales: (CAM-ICU), CAM-ICU-7, Critical Care Pain Observation Tool (CPOT), Richmond Agitation-Sedation Scale (RASS), Facial Anxiety Scale (FAS), PRE-DELIRIC model, and Glasgow Coma Scale (GCS) - Intervention twice a day for 5 days	Significant decreases were found in the severity of delirium and pain and the level of sedation and anxiety	Musical intervention can be used as a nursing intervention to control delirium, pain, the need for sedation, and anxiety in intensive care patients
		12	12			
Chlan et al., 2019 [26]	Economic evaluation of a patient-directed music intervention for ICU patients receiving mechanical ventilatory support	373 adult ICU patients receiving mechanical ventilation for acute respiratory failure		- Randomized controlled trial - Patients receiving the experimental PDMI, MP3 player, noise-canceling headphones, and music tailored to individual preferences	Savings in intensive care unit costs demonstrated; reduction in the costs of sedating drug requirements	Patient-directed music intervention is cost-effective for reducing anxiety in mechanically ventilated ICU patients
Cooke et al., 2010 [27]	To identify the effect of music on discomfort experienced by ICU patients during the turning procedure	17 postoperative patients		- Single-blind randomized cross-over design - Music of the participant’s choice listened to on earphones with a portable CD player - Listening to music with measuring discomfort and anxiety 15 min before and during the turning procedure - CG: earphones without playing music	No differences between study and control groups	No data
		10	7			
Chiasson et al., 2013 [28]	To investigate the effect of live harp music on individual patients	100 patients from the academic ICU		- Case-control design - 10 min live, spontaneous harp session played by a professional harpist - Measurements were made between 10 a.m. and 3 p.m., before and after the intervention - The CG relaxed for 10 min	No significant difference between the study and control group in physiological parameters	No data
		50	50			
Browning et al., 2020 [29]	To explore the association between therapeutic music listening as a nursing intervention for patients in the ICU and the proportion of time the patients were considered to have delirium	6 patients on mechanical ventilation		- Pilot study - 60 min therapeutic music listening twice a day - The ability to listen to three types of music in bed	The study group spent more time alert and calm than agitated. The study group also experienced less proportion of time with documented ICU delirium than the CG did	Therapeutic music listening may be used as one of the nursing interventions. It requires no additional training for staff
		3	3			
Jawaharani et al., 2020 [30]	To study the effect of music therapy as an adjunct	120 critically ill patients from an ICU		- Cross-sectional interventional study - 20 min of Mozart’s music three times a day over five days - Measurements were made on the first and fifth day - The CG had the usual care	Better scores for the Glasgow Coma Scale, heart rate, blood pressure, and the Hamilton Anxiety Scale in the study group	No data
		60	60			
Cousin et al., 2022 [31]	To examine the perception of MT by children’s parents	Parents of children from a pediatric ICU 19 X		- Retrospective cohort study summarizing the results - Patients: 25–60 min of personalized MT - MT was conducted by a certified music therapist using different instruments - Parents: assessing the impact of MT in questionnaires	Parents thought that MT helped their child during hospitalization.	Music therapy intervention may be an alternative way to support children’s and parents’ physical well-being
Yekefallah et al., 2021 [32]	The effects of musical stimulation on the level of consciousness among patients with head trauma hospitalized in intensive care units	54 patients with HT		- A randomized control trial - Fifteen-minute musical stimulation once daily for seven consecutive days - Using an MP3 player and headphones - Headphones were silent for 15 min without receiving any musical stimulation once daily for seven consecutive days - The Glasgow Coma Scale and the Richmond Agitation-Sedation Scale were used	- Improving awareness (LOC) through effective music intervention in HT patients - Music intervention can facilitate neurogenesis and neuroplasticity by increasing the level of the neurotrophic factor and can improve attention and cognitive function.	Music therapy can be used as a simple and non-expensive intervention to improve the clinical conditions of patients
		27	27			
Heiderscheit et al., 2022 [7]	Analysis of peferred music of mechanically ventilated intensive care unit patients enrolled	126 mechanically ventilated patients		- A randomized controlled trial - Patient-directed music (PDM) - 12 genres of patients self-identified as preferred with specific groups and artists requested for music listening	The effectiveness of the intervention is influenced by the choice of music	Music therapy can be used as a simple and non-expensive intervention to improve the clinical conditions of patients
Messika et al., 2016 [9]	Effect of a musical intervention on tolerance and efficacy of non-invasive ventilation in the ICU	99 adult patients (≥18 years of age)		- Study protocol for a randomized controlled trial - Sight and hearing isolation during all NIV sessions - Change in respiratory comfort (measured with a digital visual scale) before the initiation and after 30 min of the first NIV session - Respiratory and cardiovascular parameters during NIV sessions, the percentage of patients requiring endotracheal intubation, day-90 anxiety/depression and health-related quality of life, post-trauma stress induced by NIV, and the overall assessment of NIV - The observation period is 90 days - The musical intervention session lasts 30 min - Richmond Agitation-Sedation Scale, Hospital Anxiety and Depression Scale (HADS)		Non-pharmacological therapy that can affect patient perceived stress in an intensive care unit
		33	33			
Han et al., 2010 [18]	Effects of music intervention on physiological stress response and anxiety level of mechanically ventilated patients in China	137 mechanically ventilated patients in an ICU		- A randomized placebo-controlled trial - Music listening group, headphone group, control group - Chinese version of the Spielberger State-Trait Anxiety Scale - Physiological parameters (heart rate, respiratory rate, saturation of oxygen, and blood pressure) - 30 min session - Personal preference as to music choice	Significant reduction in physiological stress response and an increase in heart rate and respiratory rate in the music-listening group; reduction in anxiety in the music-listening and headphones group but not in the control group	Music can be a non-pharmacological intervention and supplement in the nursing care of mechanically ventilated patients
		Intervention group *n* = 44 Placebo group *n* = 44	49			

## Data Availability

The authors declare that the data of this research are available from the corresponding author upon request.

## Abbreviations

AMTA	The American Music Therapy Association
BP	Blood Pressure
CAM-ICU	Confusion Assessment Method in Intensive Care Unit
CAM-ICU-7	Confusion Assessment Method in Intensive Care Unit 7
CG	Control Group
CPOT	Critical Care Pain Observation Tool
DBP	Diastolic Blood Pressure
FAS	Facial Anxiety Scale
GCS	Glasgow Coma Scale
HADS	Hospital Anxiety and Depression Scale
HR	Heart Rate
HT	Head Trauma
ICU	Intensive Care Unit
LOC	Level of Consciousness
MAR	Music-Assisted Relaxation
MT	Music Therapy
NIV	Non-invasive ventilation
NRS	Numeric Rating Scale
PDM	Patient-Directed Music
PDMI	Patient-Directed Music Intervention
PDMT	Patient-Direct Music Therapy
PHQ-9	Patient Health Questionnaire-9
PTSD	The Post-Traumatic Stress Disorder
RASS	Richmond Agitation-Sedation Scale
SBP	Systolic Blood Pressure
VAS	Visual Analogue Scale
